# Supplementary material for: Single cell tracing of Pomc neurons reveals recruitment of ‘Ghost’ subtypes with atypical identity in a mouse model of obesity
Source: Nat Commun. 2024 Apr 24;15:3443. doi: 10.1038/s41467-024-47877-2 (PMC11043070; doi:10.1038/s41467-024-47877-2)
Supplement: Supplementary file 1 — Supplementary Information [file 41467_2024_47877_MOESM1_ESM.pdf]

Single cell tracing of Pomc neurons reveals recruitment of ‘Ghost’ subtypes with atypical identity in a mouse model of obesity

Stéphane Leon[1], Vincent Simon[1], Thomas H Lee[1], Lukas Steuernagel[2], Samantha Clark[1], Nasim Biglari[2], Thierry Lesté-Lasserre[1], Nathalie Dupuy[1], Astrid Cannich[1], Luigi Bellocchio[1], Philippe Zizzari[1], Camille Allard[1], Delphine Gonzales[1], Yves Le Feuvre[1], Emeline Lhuillier[3], Alexandre Brochard[1], Jean Charles Nicolas[1], Jérémie Teillon[4], Macha Nikolski[5,6], Giovanni Marsicano[1], Xavier Fioramonti[7], Jens C. Brüning[2,8,9,10,11], Daniela Cota[1], Carmelo Quarta[1#]

[1]University of Bordeaux, INSERM, Neurocentre Magendie, U1215, F-33000, Bordeaux, France.

[2]Department of Neuronal Control of Metabolism, Max Planck Institute for Metabolism Research, Cologne, Germany.

[3]University of Toulouse III Paul Sabatier, INSERM, Institut des Maladies Métaboliques et Cardiovasculaires, U1297, 31400, France; GeT-Santé, Plateforme Génome et Transcriptome, GenoToul, Toulouse, France

[4]University of Bordeaux, CNRS, INSERM, BIC, US4, UAR 3420, F-33000 Bordeaux, France

[5]University of Bordeaux, Bordeaux Bioinformatics Center, Bordeaux, France.

[6]University of Bordeaux, CNRS, IBGC UMR 5095, Bordeaux, France

[7]University of Bordeaux, INRAE, Bordeaux INP, NutriNeuro, UMR 1286, F-33000, Bordeaux, France.

[8]Center for Endocrinology, Diabetes and Preventive Medicine (CEDP), University Hospital Cologne, Cologne, Germany.

[9]Excellence Cluster on Cellular Stress Responses in Aging Associated Diseases (CECAD) University of Cologne, Cologne, Germany.

[10]Center for Molecular Medicine Cologne (CMMC), University of Cologne, Cologne, Germany.

[11]National Center for Diabetes Research (DZD), Neuherberg, Germany.

#Correspondance: carmelo.quarta@inserm.fr

Table S1

Samples	Eef1a1	Pomc 1	Pomc 2	Pomc 3	Pomc 4	Pomc 5
Ghost 1	15.05	19.92	19.93	19.89	19.89	19.79
Ghost 2	13.88	13.88	14.04	14.02	14.07	14.01
Ghost 3	16.88	23.73	23.7	13.66	23.65	23.62
Ghost 4	14.73	16.7	16.73	16.71	16.64	17.86
Ghost 5	14.33	14.44	14.5	14.49	14.51	14.34
VMH 1	16.79	-	-	-	-	-
VMH 2	15.13	-	-	-	-	-
Intrapipette ctrl	-	-	-	-	-	-
RM ctrl	-	-	-	-	-	-
Ctrl +	14.59	23.51	23.57	23.53	23.17	23.55
Preamp ctrl	-	-	-	-	-	-
qPCR ctrl	-	-	-	-	-	-

Table S1. Specific *Pomc* mRNA signal can be detected in Ghost neurons.
After obtaining *ex-vivo* living brain slices from male $Pomc^{CreERT2}$;tdTomato;Pomc-eGFP mice (n=2) 2 weeks after adult onset (12-week-old mice) administration of TAM, we aspirated the cytosolic cell content from 5 different Ghost cells, which were identified on the basis of positivity for the tdTomato signal and absence of endogenous eGFP expression. We then performed single cell qPCR of the isolated mRNAs. Ct values for Pomc and the housekeeping gene (Eef1a1) are given in the table. Pomc expression was analyzed in 5 replicates. Negative controls were included prior to cDNA synthesis and pre-amplification to exclude potential mRNA contamination during cytosolic aspiration of the target cell, including intrapipette solution (intrapipette ctrl) and reaction mix (RM ctrl). Hypothalamic mRNA from control mice was used as a positive control. Negative controls were included for both preamplification (preamp ctrl) and qPCR reactions (qPCR ctrl). The specificity of the amplification products in Ghost cells was confirmed by DNA sequencing.

Supplementary file 1

Ghost 1 sense
>EF71943945_EF71943945
GGCCGGTGCTGGCCTCCTGCTTCAGACCTCCATAGATGTGTGGAGCTGGTGCCTGGAGAGCAGCCAGTGCCAGGA
CCTCACCACGGAGAGCAACCTGCTGGCTTGCATCCGGGCTTGCAAACTCGACCTCTCGCTGGAGACGCCCGTGTT
TCCTGGCAACGGAGATGAACAGCCCCTGACTGAAAACCCCCGGAAGTACGTCATGGGTCACTTCCGCTGGGACCG
CTTCGGCCCCAGGAACAGCAGCAGT

Ghost 1 antisense
>EF71943952_EF71943952
CGGGAGGCCGCGGAAGTGACCATGACGTACTTCCGGGGGTTTTCAGTCAGGGGCTGTTCATCTCCGTTGCCAGGA
AACACGGGCGTCTCCAGCGAGAGGTCGAGTTTGCAAGCCCGGATGCAAGCCAGCAGGTTGCTCTCCGTGGTGAGG
TCCTGGCACTGGCTGCTCTCCAGGCACCAGCTCCACACATCTATGGAGGTCTGAAGCAGGAGGGCCAGCAACAGG
GCCCCTGAGCGACTGTAGCAGAATCTCGGCATAAC

Ghost 2 sense
>EF71943948_EF71943948
GCTGTTGCTGGCCTCCTGCTTCAGACCTCCATAGATGTGTGGAGCTGGTGCCTGGAGAGCAGCCAGTGCCAGGAC
CTCACCACGGAGAGCAACCTGCTGGCTTGCATCCGGGCTTGCAAACTCGACCTCTCGCTGGAGACGCCCGTGTTT
CCTGGCAACGGAGATGAACAGCCCCTGACTGAAAACCCCCGGAAGTACGTCATGGGTCACTTCCGCTGGGACCGC
TTCGGCCCCAGGAACAGCAGCAGTGCTGGCAAGA

Ghost 2 antisense
>EF71943955_EF71943955
GACCCATGACGTACTTCCGGGGGTTTTCAGTCAGGGGCTGTTCATCTCCGTTGCCAGGAAACACGGGCGTCTCCA
GCGAGAGGTCGAGTTTGCAAGCCCGGATGCAAGCCAGCAGGTTGCTCTCCGTGGTGAGGTCCTGGCACTGGCTGC
TCTCCAGGCACCAGCTCCACACATCTATGGAGGTCTGAAGCAGGAGGGCCAGCAACAGGGCCCCTGAGCGACTGT
AGCAGAATCTCGGC

Ghost 3 sense
>EF71943949_EF71943949
AGACCTCCATAGATGTGTGGAGCTGGTGCCTGGAGAGCAGCCAGTGCCAGGACCTCACCACGGAGAGCAACCTGC
TGGCTTGCATCCGGGCTTGCAAACTCGACCTCTCGCTGGAGACGCCCGTGTTTCCTGGCAACGGAGATGAACAGC
CCCTGACTGAAAACCCCCGGAAGTACGTCATGGGTCACTTCCGCTGGGACCGCTTCGGCCCCAGGAACAGCAGCA
GTGCTGGCAATTATG

Ghost 3 antisense
>EF71943956_EF71943956
AGTGACCCATGACGTACTTCCGGGGGTTTTCAGTCAGGGGCTGTTCATCTCCGTTGCCAGGAAACACGGGCGTCT
CCAGCGAGAGGTCGAGTTTGCAAGCCCGGATGCAAGCCAGCAGGTTGCTCTCCGTGGTGAGGTCCTGGCACTGGC
TGCTCTCCAGGCACCAGCTCCACACATCTATGGAGGTCTGAAGCAGGAGGGCCAGCAACAGGGCCCCTGAGCGAC
TGTAGCAGAATCTCGGC

Ghost 4 sense
>EF71943950_EF71943950
ACCGGCCGTTGCTGGCCTCCTGCTTCAGACCTCCATAGATGTGTGGAGCTGGTGCCTGGAGAGCAGCCAGTGCCA
GGACCTCACCACGGAGAGCAACCTGCTGGCTTGCATCCGGGCTTGCAAACTCGACCTCTCGCTGGAGACGCCCGT
GTTTCCTGGCAACGGAGATGAACAGCCCCTGACTGAAAACCCCCGGAAGTACGTCATGGGTCACTTCCGCTGGGA
CCGCTTCGGCCCCAGGAACAGCAGCAGTGCTGGCAAAGA

Ghost 4 antisense
>EF71943957_EF71943957
TGACCCATGACGTACTTCCGGGGGTTTTCAGTCAGGGGCTGTTCATCTCCGTTGCCAGGAAACACGGGCGTCTCC
AGCGAGAGGTCGAGTTTGCAAGCCCGGATGCAAGCCAGCAGGTTGCTCTCCGTGGTGAGGTCCTGGCACTGGCTG
CTCTCCAGGCACCAGCTCCACACATCTATGGAGGTCTGAAGCAGGAGGGCCAGCAACAGGGCCCCTGAGCGACTG
TAGCAGAATCTCGGCATAAGT

```
Ghost 5 sense
>EF71943951_EF71943951
CGGCGGTGGCTGGCCTCCTGCTTCAGACCTCCATAGATGTGTGGAGCTGGTGCCTGGAGAGCAGCCAGTGCCAGG
ACCTCACCACGGAGAGCAACCTGCTGGCTTGCATCCGGGCTTGCAAACTCGACCTCTCGCTGGAGACGCCCGTGT
TTCCTGGCAACGGAGATGAACAGCCCCTGACTGAAAACCCCCGGAAGTACGTCATGGGTCACTTCCGCTGGGACC
GCTTCGGCCCCAGGAACAGCAGCAGTGCTGGCAAGAA

Ghost 5 antisense
>EF71943958_EF71943958
GACCCATGACGTACTTCCGGGGGTTTTCAGTCAGGGGCTGTTCATCTCCGTTGCCAGGAAACACGGGCGTCTCCA
GCGAGAGGTCGAGTTTGCAAGCCCGGATGCAAGCCAGCAGGTTGCTCTCCGTGGTGAGGTCCTGGCACTGGCTGC
TCTCCAGGCACCAGCTCCACACATCTATGGAGGTCTGAAGCAGGAGGGCCAGCAACAGGGCCCCTGAGCGACTGT
AGCAGAATCTCGGC
```

Supplementary file 1

Sequencing analysis of the amplified Pomc transcripts from Table S1. We confirmed 99% sequence homology with Pomc variants using Blast alignment tool.

Table S2

Genotyping primers

Mouse line	Primer	Sequence (5'-3')	Expected fragment	Lenght
Pomc-CreER^{T2}	Cre-F	GCGGTCTGGCAGTAAAAACTATC	CreERt2	105 pb
	Cre-R	GTGAAACAGCATTGCTGTCACTT		
Pomc-eGFP	GFP-F	GCACGACTTCTTCAAGTCCGCCATGCC	GFP	280 pb
	GFP-R	GCGGATCTTGAAGTTCACCTTGATGCC		
Ai6	Ai6-WT-F	AAGGGAGCTGCAGTGGAGTA	Rosa wt	297 pb
	Ai6-WT-R	CCGAAAATCTGTGGGAAGTC		
	Ai6-mut-F	GGCATTAAAGCAGCGTATCC	WPRE	199 pb
	Ai6-mut-R	AACCAGAAGTGGCACCTGAC	ZsGreen	
AI14	Ai14-WT-F	AAGGGAGCTGCAGTGGAGTA	Rosa wt	297 pb
	Ai14-WT-R	CCGAAAATCTGTGGGAAGTC		
	Ai14-mut-F	GGCATTAAAGCAGCGTATCC	WPRE	215 pb
	Ai14-mut-R	CTGTTCCTGTACGGCATGG	tdTomato	

qPCR primers

Gene	GenBank ID	Forward Sequence (5'-3')	Reverse Sequence (5'-3')
Pomc	NM_001278581	ATGCCGAGATTCTGCTACAGT	GCCAGCACTGCTGCTGTTC
Eef1a1	NM_010106	CCATGTGTGTTGAGAGCTTCT	GCAACTGTCTGCCTCATGTCA

Table S2. List of primers used for mouse genotyping. sc-qPCR pre-amplification and qPCR

Figure S1 related to figure 1

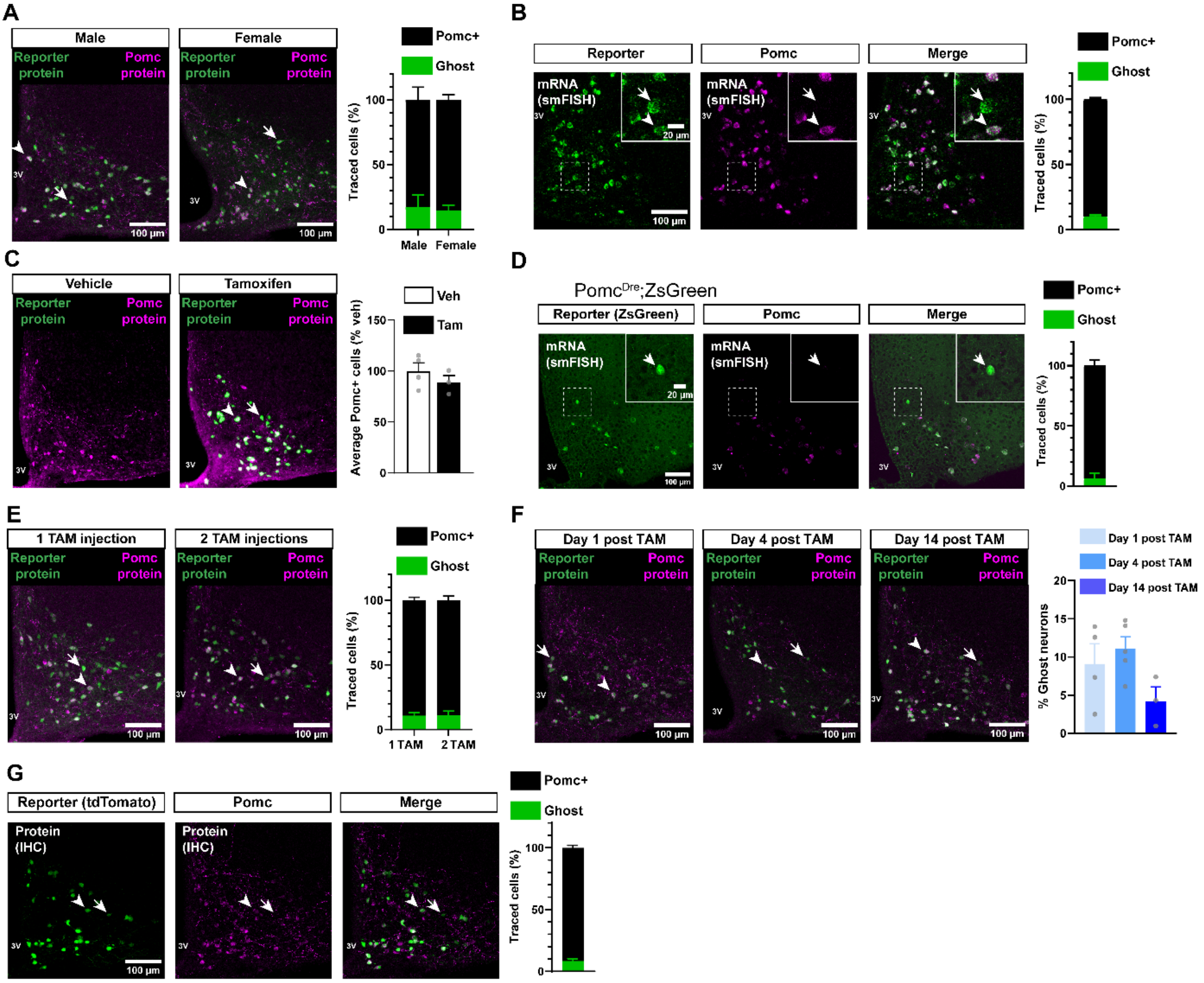

Figure S1. Related to Figure 1. Lineage tracing of POMC neurons in adult chow-fed mice reveals subtypes with atypical molecular identity.

(A) Detection and quantification of the % of Pomc+ (arrowhead) and Ghost neurons (arrow) in Pomc^{CreERT2};tdTomato;Pomc-eGFP male (n=4) and female (n=4) mice 2 weeks after TAM administration in12-week-old mice by IHC.

(B) Detection and quantification of the % of Pomc+ (arrowhead) and Ghost neurons (arrow) in Pomc^{CreERT2};ZsGreen male mice 2 weeks after after TAM administration in 12-week-old mice (n=5). We analyzed the number of reporter cells (ZsGreen) positive (Pomc+) or negative (Ghost) for *Pomc* mRNA by smFISH.

(C) Left: Representative microscopic images of IHC against Pomc protein and ZsGreen reporter in 12-week-old Pomc^{CreERT2};ZsGreen male mice 2 weeks after vehicle (n=4) or TAM (150mg/kg for 5 days, n=3) administration. Right: Quantification of the relative number of ARC Pomc+ cells in vehicle vs. TAM-treated reporter mice.

(D) Detection and quantification of the % of Pomc+ (arrowhead) and Ghost neurons (arrow) in 12-week-old Pomc^{Dre};ZsGreen male mice (n=3). The number of reporter cells (*ZsGreen* mRNA) positive (Pomc+) or negative (Ghost) for *Pomc* mRNA was analyzed by smFISH.

(E) Left: Representative IHC images of Pomc protein and endogenous tdTomato reporter in $Pomc^{CreERT2}$;tdTomato male mice treated with 1 cycle (n=4) or 2 repeated cycles (n=4) of TAM administration (150 mg/kg for 5 days). Samples were collected 2 weeks after TAM administration (12-week-old). Right: relative quantification of Pomc+ and Ghost neurons.

(F) Left: Representative IHC images of Pomc protein and endogenous tdTomato reporter in $Pomc^{CreERT2}$;tdTomato male mice at day 1 (n=4), 4 (n=5) and 14 (n=3) after tamoxifen administration (150 mg/kg for 5 days in 12-week-old mice). Right: relative quantification of Pomc+ and Ghost neurons.

(G) Left: Representative microscopic images of IHC against Pomc protein and endogenous tdTomato reporter in 5-week-old $Pomc^{CreERT2}$;tdTomato male mice (n=5). Samples were collected 2 weeks after TAM administration. Right: relative quantification of Pomc+ and Ghost neurons.

IHC: immunohistochemistry, FISH: fluorescence in situ hybridisation, smFISH: single molecule fluorescence in situ hybridisation. Data in A-G are mean ± s.e.m from 3 independent experiments. n indicates the individual biological values. Arrowheads define Pomc+ neurons, whereas arrows define Ghost neurons. Source data are provided as a Source Data file.

Figure S2 related to figure 1

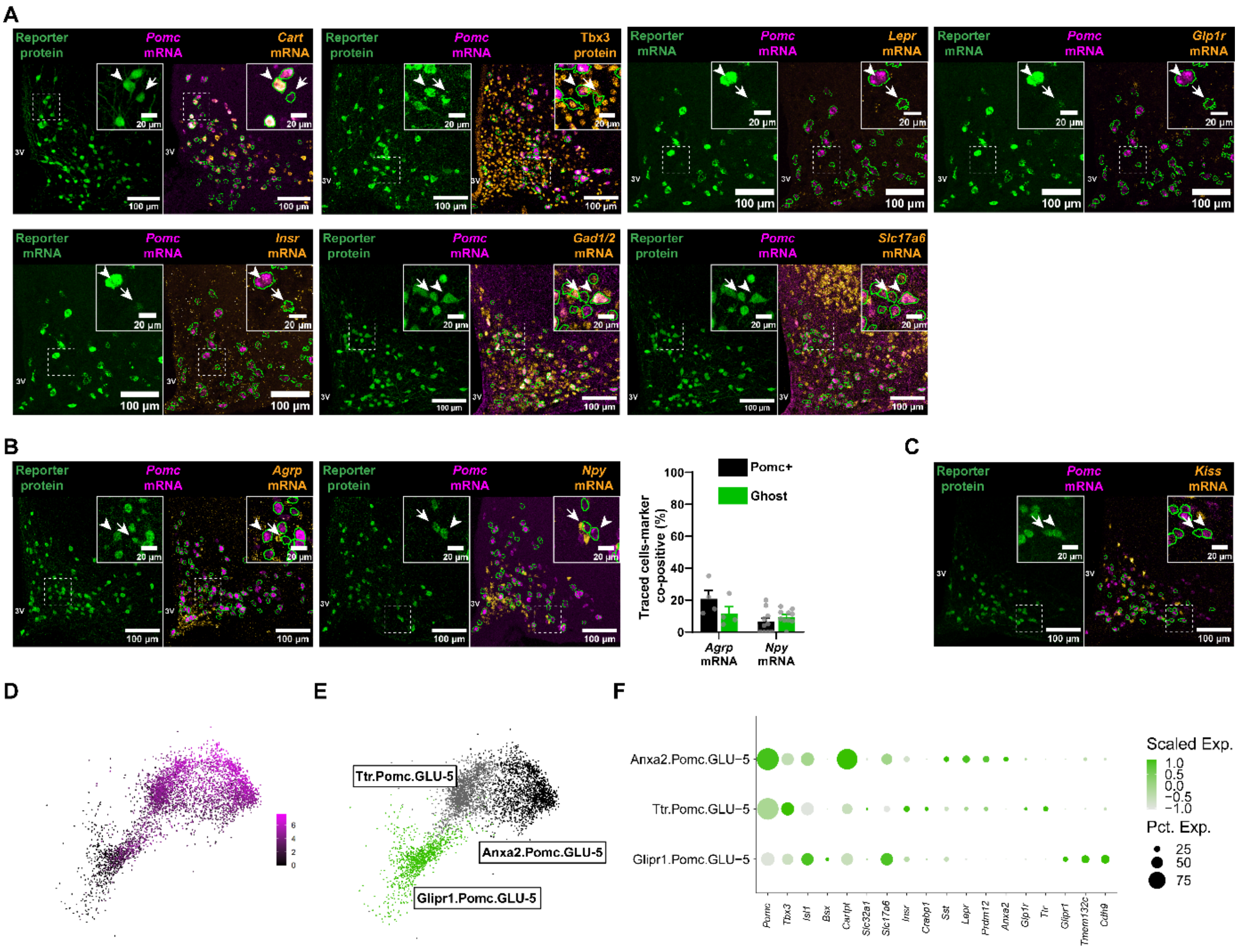

Figure S2. Related to Figure 1. Lineage tracing of POMC neurons in chow-fed mice reveals subtypes with atypical molecular identity.

(A) Representative images relative the analysis shown in main Figure 1D.

(B) Representative images and quantification of the % of Pomc+ and Ghost neurons co-expressing detectable mRNA levels of *AgRP* (n=4) or *Npy* (n=10) in Pomc^{CreERT2};ZsGreen male mice 2 weeks after adult onset (12-week-old mice) administration of TAM, as assessed by FISH (*Pomc*, *AgRP* and *Npy*) and IHC (ZsGreen).

(C) Representative image showing no colocalization between traced cells with *Kiss* mRNA in Pomc^{CreERT2};tdTomato male mice (n=4) 2 weeks after adult onset (12-week-old mice) administration of TAM, as assessed by FISH (*Pomc* and *Kiss*) and IHC (tdTomato).

(D-E) UMAP plots of Pomc neurons in the hypothalamus single-cell reference atlas HypoMap. Log-normalized *Pomc* expression in each cell (D) and subcluster identity of each cell (E) are shown.

(F) Dotplot of marker gene expression in the Pomc subclusters of HypoMap. The size of the dots corresponds to the percentage of cells expressing a respective gene in each cluster and the color intensity of the dots to the average scaled expression.

Data in B are mean ± s.e.m. from 3 independent experiments n indicates the individual biological values. In A, B, C, arrowheads define Pomc+ neurons, whereas arrows define Ghost neurons. Source data are provided as a Source Data file.

Figure S3 related to figure 2

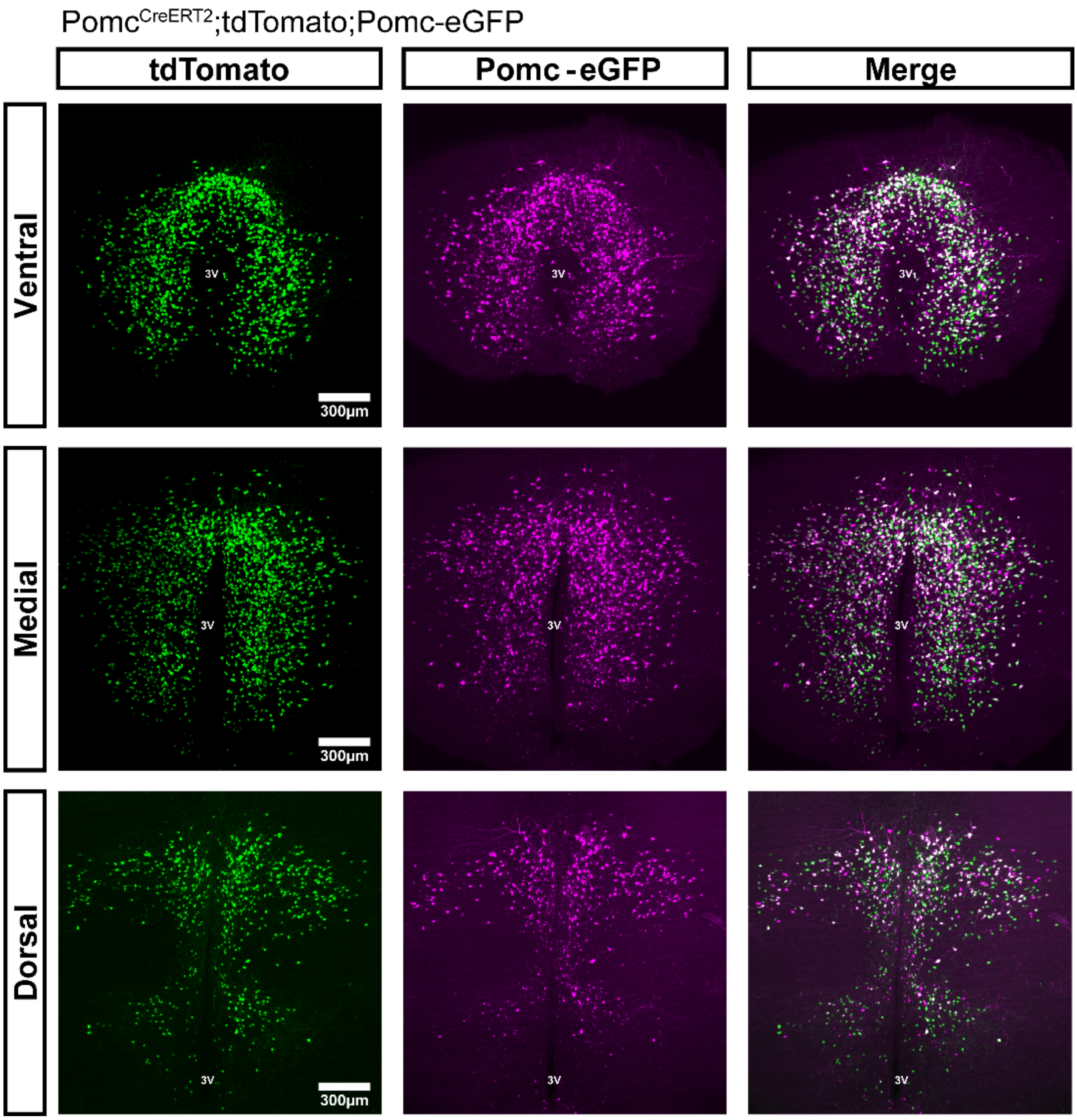

Figure S3. Related to Figure 2. Ghost neurons show a distinct spatial distribution under chow-diet. Representative images of CUBIC transparentised brains of Pomc^{CreERT2};tdTomato;Pomc-eGFP male mice (n=3), obtained 2 weeks after adult onset administration of tamoxifen to 12-week-old animals. tdTomato (green) and Pomc-eGFP (purple) signals are shown in different regions of the ARC (ventral, medial and caudal).

Figure S4 related to figure 3

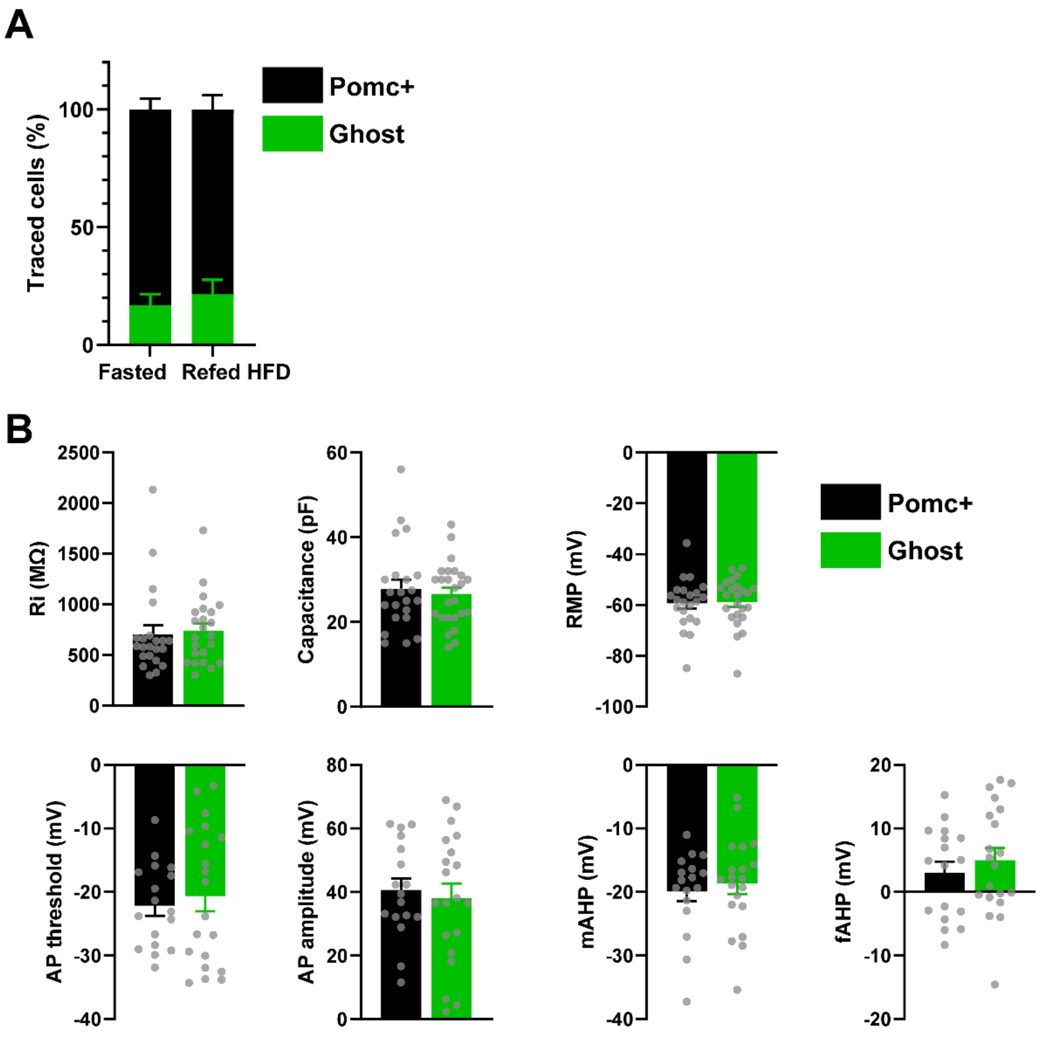

Figure S4. Related to Figure 3. Ghost cells have atypical sensitivity to nutritional and hormonal cues

(A) Quantification of the percentage of Pomc+ and Ghost neurons in Pomc^{CreERT2};tdTomato male mice challenged with fasting (24 hours, n=4) or fasting followed by refeeding (2 hours, n=4) with respect to Figure 3A-B. Twelve-week-old mice were exposed to food cues 2 weeks after tamoxifen administration.

(B) Basal electrophysiological properties of Pomc+ and Ghost cells before leptin or insulin application in relation to the experiment described in Figure 3E and 3F. Ri: input resistance; AP: action potential; mAHP: medium-duration afterhyperpolarization; fAHP: fast afterhyperpolarization.

Data in (A) are mean ± s.e.m from 2 independent experiments. Data in (B) are mean ± s.e.m from 3 independent experiments. n indicates the individual biological values. Source data are provided as a Source Data file.

Figure S5 related to figure 4

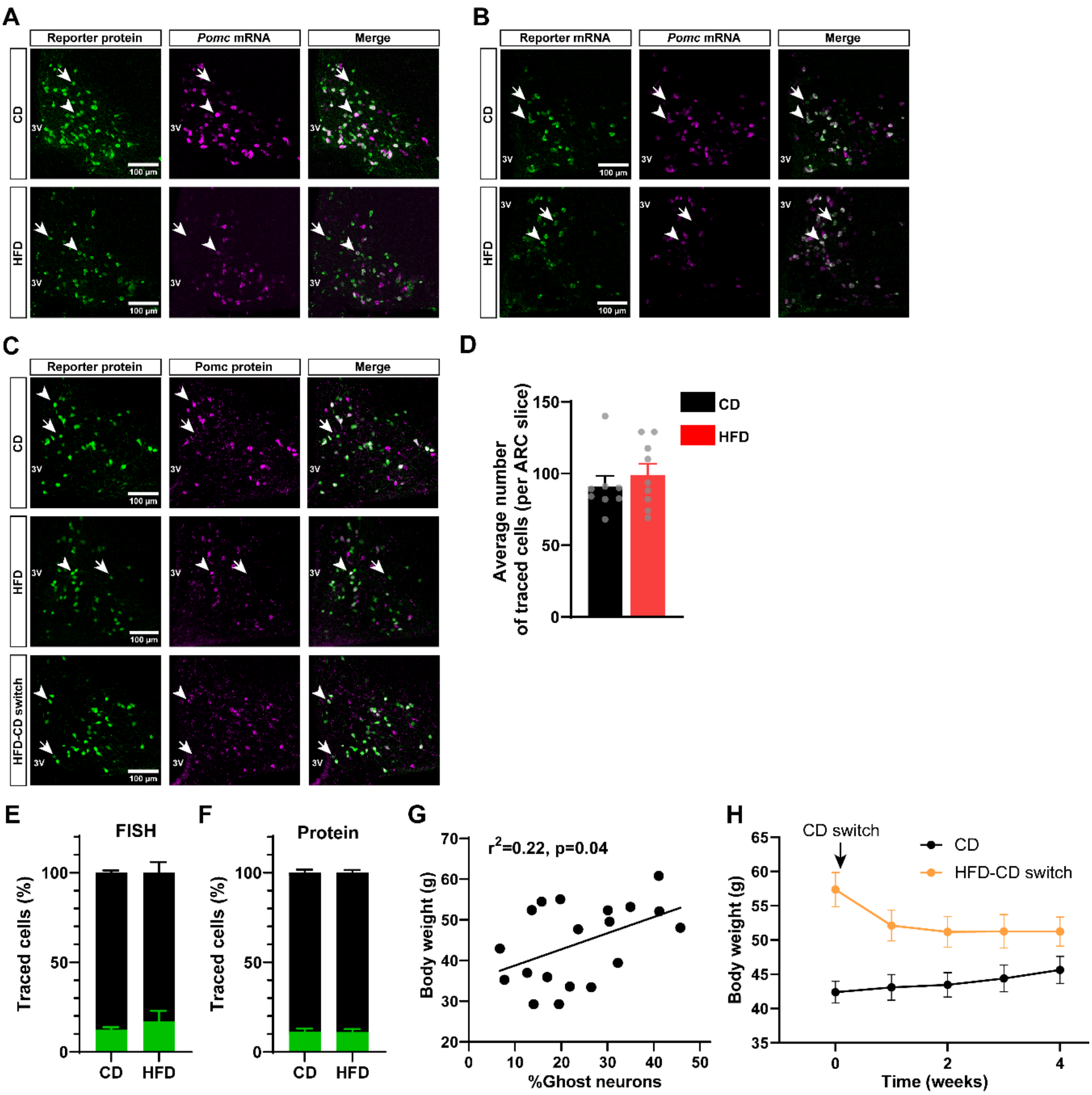

Figure S5. Related to Figure 4. Ghost cell recruitment in HFD-induced obesity

(A-C) Representatives images relative the analysis shown respectively in main Figure 4B-D.

(D) Total average number of reporter (ZsGreen) positive cells per ARC section in $Pomc^{CreERT2}$;ZsGreen male mice fed on CD (n=8) or HFD (n=9) for 6 months, from the data in Figure 4B.

(E) Quantification of Pomc+ and Ghost neurons by FISH. in $Pomc^{CreERT2}$;ZsGreen male mice fed with CD (n=10) or HFD (n=6) for 3 months. Mice were treated with tamoxifen at 12 weeks of age and exposed to HFD or CD 2 weeks later.

(F) Quantification of Pomc+ and Ghost neurons by IHC in $Pomc^{CreERT2}$;tdTomato male mice fed with CD (n=10) or HFD (n=12) for 3 months. Mice were treated with tamoxifen at 12 weeks of age and exposed to HFD or CD 2 weeks later.

(G) Linear regression analysis between Ghost neuron numbers (%) in DIO mice fed with HFD for 6 months and body weight (n=19).

(H) Body weight measurements of control CD-fed $Pomc^{CreERT2}$;tdTomato male mice (n=3) compared to HFD (6 months) mice or HFD-CD switch mice. Mice were treated with tamoxifen at 12 weeks of age and exposed to the different diets 2 weeks after tamoxifen treatment.

CD: chow diet, HFD: high fat diet, IHC: immunohistochemistry. Data in D-F are mean ± s.e.m. from 3 independent experiments. n indicates the individual biological values. Data in G presents statistical significance using linear regression of values pooled from 3 independent experiments. In A, B, C, arrowheads define Pomc+ neurons, whereas arrows define Ghost neurons. Source data are provided as a Source Data file.

Figure S6 related to figure 4

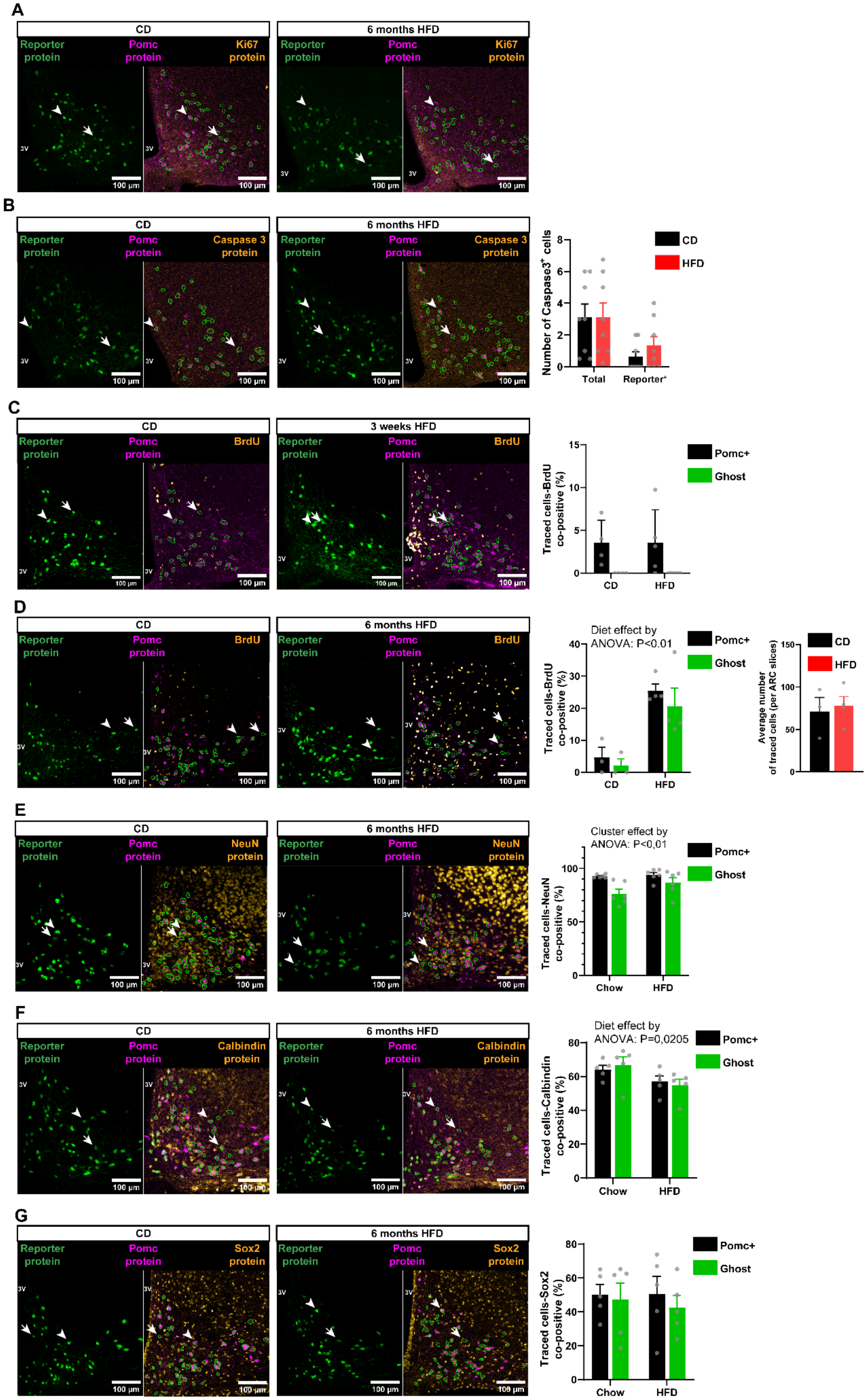

Figure S6. Related to Figure 4. Ghost cell recruitment in HFD-induced obesity

(A) Representative IHC images of Ki67 in Pomc+ and Ghost neurons in $Pomc^{CreERT2}$;tdTomato male mice fed with CD (n=4) or HFD (n=4) for 6 months.

(B) Representative IHC images and quantification of the total average number of arcuate nucleus cells or reporter-positive cells expressing cleaved caspase 3 in $Pomc^{CreERT2}$;ZsGreen male mice fed with CD (n=8) or HFD (n=8) for 6 months.

(C) Representative IHC images and quantification of the % of Pomc+ and Ghost neurons positive for BrdU in $Pomc^{CreERT2}$;tdTomato;Pomc-eGFP male mice fed with CD (n=4) or HFD (3 weeks, n=5) and receiving continuous ICV infusion of BrdU for 3 weeks.

(D) Representative IHC images and quantification of the % of Pomc+ and Ghost neurons positive for BrdU in $Pomc^{CreERT2}$;tdTomato;Pomc-eGFP male mice fed with CD (n=3) or HFD (6 months, n=4) and receiving continuous ICV infusion of BrdU from month 3 of HFD vs CD administration until month 6.

(E) Representative IHC images and quantification of the of the % of NeuN positive Pomc+ and Ghost neurons in $Pomc^{CreERT2}$;tdTomato male mice fed with CD (n=6) or HFD (n=6) for 6 months after tamoxifen administration.

(F) Representative IHC images and quantification of the of the % of and Calbindin-D28K positive Pomc+ and Ghost neurons in $Pomc^{CreERT2}$;tdTomato male mice fed with CD (n=5) or HFD (n=5) for 6 months after tamoxifen administration.

(G) Representative IHC images and quantification of the of the % of and Sox2 positive Pomc+ and Ghost neurons in $Pomc^{CreERT2}$;tdTomato male mice fed with CD (n=5) or HFD (n=5) for 6 months after tamoxifen administration.

IHC: immunohistochemistry, CD: chow diet, HFD: high fat diet, Ki67: antigen kiel 67, Sox2: SRY-Box transcription factor 2, BrdU: Bromodeoxyuridine. All mice were treated with tamoxifen at 12 weeks of age and exposed to the different diets 2 weeks after TAM treatment. Data in B-G are mean ± s.e.m from 3 independent experiments. n indicates the individual biological values. Statistical analysis in D-F shows significance of subpopulation or diet factor after two-way ANOVA. Arrowheads define Pomc+ neurons, arrows indicate Ghost neurons. Source data are provided as a Source Data file.

Figure S7 related to figure 4

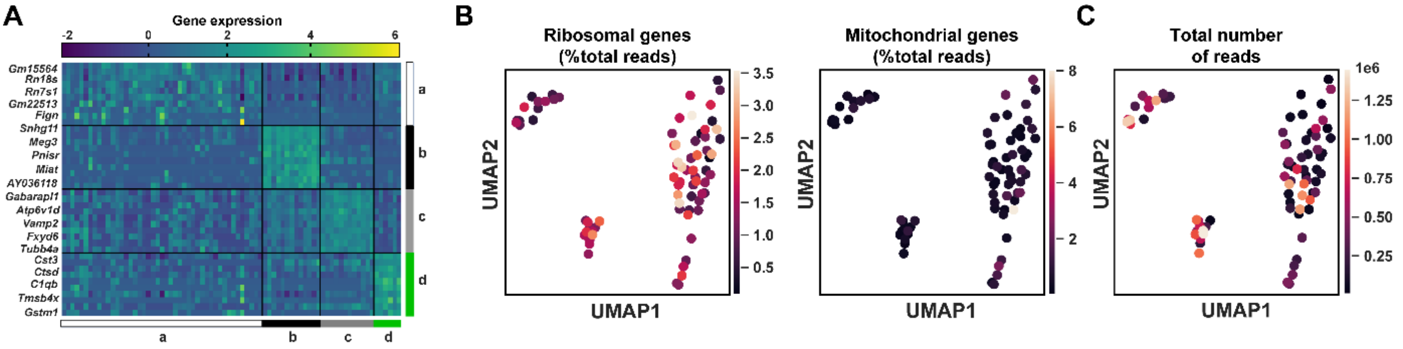

Figure S7. Analysis of Ghost neurons by Patch-seq

(A) Heatmap showing the gene expression defining the 4 clusters identified in Figure 4E by Patch-Seq.

(B) UMAP representation of the cluster analysis of the molecular profile of the 76 single-tracked neurons analyzed by Patch-Seq. We show levels of expression (percentage of reads) for ribosomal and mitochondrial markers.

(C) UMAP representation of the cluster analysis of the molecular profile of the 76 single-tracked neurons by Patch-Seq. We show total number of reads relative to each single cell identified within the different clusters. n indicates the number of biologically independent samples examined. Source data are available in GEO n° GSE261715.
