# Supplementary material for: Single cell tracing of Pomc neurons reveals recruitment of ‘Ghost’ subtypes with atypical identity in a mouse model of obesity
Source: Nat Commun. 2024 Apr 24;15:3443. doi: 10.1038/s41467-024-47877-2 (PMC11043070; doi:10.1038/s41467-024-47877-2)
Supplement: Supplementary file 4 — Reporting Summary [file 41467_2024_47877_MOESM4_ESM.pdf]

nature portfolio

Corresponding author(s): Carmelo Quarta
Last updated by author(s): 16/03/2024

Reporting Summary

Nature Portfolio wishes to improve the reproducibility of the work that we publish. This form provides structure for consistency and transparency in reporting. For further information on Nature Portfolio policies, see our Editorial Policies and the Editorial Policy Checklist.

Statistics

For all statistical analyses, confirm that the following items are present in the figure legend, table legend, main text, or Methods section.

n/a	Confirmed	
☐	☒	The exact sample size (*n*) for each experimental group/condition, given as a discrete number and unit of measurement
☐	☒	A statement on whether measurements were taken from distinct samples or whether the same sample was measured repeatedly
☐	☒	The statistical test(s) used AND whether they are one- or two-sided<br>*Only common tests should be described solely by name; describe more complex techniques in the Methods section.*
☒	☐	A description of all covariates tested
☐	☒	A description of any assumptions or corrections, such as tests of normality and adjustment for multiple comparisons
☐	☒	A full description of the statistical parameters including central tendency (e.g. means) or other basic estimates (e.g. regression coefficient) AND variation (e.g. standard deviation) or associated estimates of uncertainty (e.g. confidence intervals)
☐	☒	For null hypothesis testing, the test statistic (e.g. *F*, *t*, *r*) with confidence intervals, effect sizes, degrees of freedom and *P* value noted<br>*Give P values as exact values whenever suitable.*
☒	☐	For Bayesian analysis, information on the choice of priors and Markov chain Monte Carlo settings
☒	☐	For hierarchical and complex designs, identification of the appropriate level for tests and full reporting of outcomes
☐	☒	Estimates of effect sizes (e.g. Cohen's *d*, Pearson's *r*), indicating how they were calculated

Our web collection on statistics for biologists contains articles on many of the points above.

Software and code

Policy information about availability of computer code

Data collection	Confocal images acquisition were performed using Leica Application Suite (v3.5.7) and a Leica DM6 CFS TCS SP8 confocal microscope equipped with an X20/1 dry objective. CUBIC cleared brains were acquired on a Ultramicroscope II light sheet microscope (LaVision Biotec, Germany) using a sCMOS camera (Andor Neo) and a 2x/0.5 objective lens. Electrophysiological data were collected using Clampex (Molecular Devices, v11.1); sc-qPCR data were generated using the LightCycler® 480 (Roche Applied Science) and the Labchip GX II (Caliper life sciences).<br>Python code used for isodensity plots generation and 3D statistical analysis (Figure 2) was provided by Jens Brüning and is available at https://github.com/bruening-lab/Heterogeneity_Scripts.<br>Cluster analysis Python code was provided by Yves Le Feuvre and is available upon request.<br>The processed single-cell RNAseq data analysed in Figure 1 and Figure S2 (HypoMap) is available online (https://www.repository.cam.ac.uk/handle/1810/340518).
Data analysis	Details are provided in the Methods section.<br>FIJI (National Institute of Health, v1.53t) for all image analysis; Microsoft Excel 365; Prism (Graphpad, v9.5) for all graph generation and statistics; Python (v3.3) for cluster analysis and isodensity plots; Electrophysiological data were analysed using Esay Electrophysiology (v2.5.2)

For manuscripts utilizing custom algorithms or software that are central to the research but not yet described in published literature, software must be made available to editors and reviewers. We strongly encourage code deposition in a community repository (e.g. GitHub). See the Nature Portfolio guidelines for submitting code & software for further information.

Data

Policy information about availability of data

All manuscripts must include a data availability statement. This statement should provide the following information, where applicable:

- Accession codes, unique identifiers, or web links for publicly available datasets
- A description of any restrictions on data availability
- For clinical datasets or third party data, please ensure that the statement adheres to our policy

Source data and representative images are provided with this paper. All other data supporting the findings of this study are available from the corresponding author upon reasonable request.
The processed single-cell RNAseq data analysed in Figure 1 and Figure S2 (HypoMap) is available online (https://www.repository.cam.ac.uk/handle/1810/340518).
The scRNA-seq data generated in this study have been deposited in the Gene Expression Omnibus (GEO) database under the accession code GSE261715

Research involving human participants, their data, or biological material

Policy information about studies with human participants or human data. See also policy information about sex, gender (identity/presentation), and sexual orientation and race, ethnicity and racism.

Reporting on sex and gender	N/A
Reporting on race, ethnicity, or other socially relevant groupings	N/A
Population characteristics	N/A
Recruitment	N/A
Ethics oversight	N/A

Note that full information on the approval of the study protocol must also be provided in the manuscript.

Field-specific reporting

Please select the one below that is the best fit for your research. If you are not sure, read the appropriate sections before making your selection.

☒ Life sciences ☐ Behavioural & social sciences ☐ Ecological, evolutionary & environmental sciences

For a reference copy of the document with all sections, see nature.com/documents/nr-reporting-summary-flat.pdf

Life sciences study design

All studies must disclose on these points even when the disclosure is negative.

Sample size	We did not specifically perform a sample size calculation for most of studies as the magnitude of effect sizes were previously unknown. In vivo studies were performed with different sample size based on animals availability and/or instructions from Ethical Authorities, in order to obtain the best statistical power possible. All n values are reported throughout the text. We used at least 3 biological replicates for each imaging experiment.
Data exclusions	No data were excluded from the analyses unless technical mistakes or uncontrollable situations occurred that could result in inaccuracy, such as improper brain dissection/sectioning, technical problems during image acquisition leading to scarce imaging quality
Replication	The main imaging endpoints of the study (changes in Ghost vs. Pomc+ neurons) were replicated in at least 3 independent cohorts of animals for the majority of experiments, with the exception of a few (Figures 3A-3D and 4D) which were independently replicated twice. The data in Figure 2 were replicated twice. All replication attempts were successful.
Randomization	For all experiments, mice were randomly assigned to different dietary or treatment groups
Blinding	Data collection and analysis were carried out in a blinded format throughout the study. In particular, neuroanatomical analysis were performed using automated FIJI macro with fixed parameters for all samples.

Reporting for specific materials, systems and methods

We require information from authors about some types of materials, experimental systems and methods used in many studies. Here, indicate whether each material, system or method listed is relevant to your study. If you are not sure if a list item applies to your research, read the appropriate section before selecting a response.

Materials & experimental systems

n/a	Involved in the study
☐	☒ Antibodies
☒	☐ Eukaryotic cell lines
☒	☐ Palaeontology and archaeology
☐	☒ Animals and other organisms
☒	☐ Clinical data
☒	☐ Dual use research of concern
☒	☐ Plants

Methods

n/a	Involved in the study
☒	☐ ChIP-seq
☒	☐ Flow cytometry
☒	☐ MRI-based neuroimaging

Antibodies

Antibodies used

Primary antibodies:
Pomc: rabbit anti-POMC (Phoenix Pharmaceuticals, H-029-30, 1:2000)
Tbx3: rabbit anti-Tbx3 (Bethyl, A303-098A, 1:500),
c-Fos: rabbit anti-c-Fos (Santa Cruz, sc-52, 1:500)
pStat3: rabbit anti-phospho STAT3 (Tyr705) (Cell Signaling, 9131, 1:500)
Ki67: mouse anti-Ki-67 (BD Biosciences, 550609, 1:500)
Caspase3: rabbit anti-cleaved caspase-3 (Asp175) (Cell Signaling, 9661, 1:500)
BrdU: rat anti-BrdU (Abcam, ab6326, 1:500)
NeuN: chicken anti-NeuN (Millipore, ABN91, 1:2000)
tdTomato: goat anti-tdTomato (Sicgen, AB8181, 1:500)
ZsGreen: guinea-pig anti-ZsGreen (Frontier Institute, Af940, 1:500)
Calbindin-D28K: rabbit anti-Calbindin-D28K (Sigma-Aldrich, C2724, 1:500)
Sox2: rabbit anti-Sox2 (EMD Millipore, AB5603, 1:500)

Secondary antibodies:
Donkey anti-rabbit IgG AF647 (Jackson Immunoresearch, 711-605-152, 1:500)
Goat anti-mouse AF488 Fab2 (Cell Signaling, 4408S, 1:500)
Goat anti-rat-Biotin (Vector Laboratories, #BA-9401, 1:500)
Donkey anti-chicken Fab2 AF647 (Jackson Immunoresearch, 703-606-155, 1:500)
Donkey anti-goat AF647 (Invitrogen, A21447, 1:500)
Donkey anti-guinea-pig AF 647 (Jackson Immunoresearch, 706-605-148, 1:500)

Validation

All commercial antibodies were validated by the manufacture (except for Tbx3) and commonly used in our lab. Information about host species, reactivity, and applications are freely available from manufacturer's websites that are reported below.

Primary antibodies:
Rabbit anti-POMC (Phoenix Pharmaceuticals, H-029-30)
https://www.phoenixpeptide.com/products/view/Antibodies/H-029-30

Rabbit anti-Tbx3 (Bethyl, A303-098A)
https://www.thermofisher.com/antibody/product/TBX3-Antibody-Polyclonal/A303-098A
Validated in Quarta et al Nature Metabolism 2019 Feb;1(2):222-23

Rabbit anti-c-Fos (Santa Cruz, sc-52)
https://scbt.com/fr/p/c-fos-antibody-4

Mouse anti-Ki-67 (BD Biosciences, 550609)
https://www.bdbiosciences.com/en-nz/products/reagents/flow-cytometry-reagents/research-reagents/single-color-antibodies-ruo/purified-mouse-anti-ki-67.550609

Rabbit anti-cleaved caspase-3 (Asp175) (Cell Signaling, 9661)
https://www.cellsignal.com/products/primary-antibodies/cleaved-caspase-3-asp175-antibody/9661

Rat anti-BrdU (Abcam, ab6326)
https://www.abcam.com/products/primary-antibodies/brdu-antibody-bu175-icr1-proliferation-marker-ab6326.html

Chicken anti-NeuN (Millipore, ABN91)
https://www.merckmillipore.com/LU/fr/product/Anti-NeuN-Antibody,MM_NF-ABN91

Goat anti-tdTomato (Sicgen, AB8181)
https://www.origene.com/catalog/antibodies/tag-antibodies/ab8181-200/tdtomato-goat-polyclonal-antibody

Guinea-pig anti-ZsGreen (Frontier Institute, Af940)

https://nittobo-nmd.co.jp/pdf/reagents/ZsGreen.pdf

Rabbit anti-Calbindin-D28K (Sigma-Aldrich, C2724)
https://www.sigmaaldrich.com/FR/fr/product/sigma/c2724

Rabbit anti-Sox2 (EMD Millipore, AB5603)
https://www.merckmillipore.com/FR/fr/product/Anti-Sox2-Antibody,MM_NF-AB5603

Secondary antibodies:
Donkey anti-rabbit IgG AF647 (Jackson Immunoresearch, 711-605-152)
https://www.jacksonimmuno.com/catalog/products/711-605-152

Goat anti-mouse AF488 Fab2 (Cell Signaling, 4408S)
https://www.cellsignal.com/products/secondary-antibodies/anti-mouse-igg-h-l-f-ab-2-fragment-alexa-fluor-488-conjugate/4408

Goat anti-rat-Biotin (Vector Laboratories, #BA-9401)
https://vectorlabs.com/products/antibodies/biotinylated-goat-anti-rat-igg-mouse-adsorbed

Donkey anti-chicken Fab2 AF647 (Jackson Immunoresearch, 703-606-155)
https://www.jacksonimmuno.com/catalog/products/703-606-155

Donkey anti-goat AF647 (Invitrogen, A21447)
https://www.thermofisher.com/antibody/product/Donkey-anti-Goat-IgG-H-L-Cross-Adsorbed-Secondary-Antibody-Polyclonal/A-21447

Donkey anti-guinea-pig AF 647 (Jackson Immunoresearch, 706-605-148)
https://www.jacksonimmuno.com/catalog/products/706-605-148

Animals and other research organisms

Policy information about studies involving animals; ARRIVE guidelines recommended for reporting animal research, and Sex and Gender in Research

Laboratory animals	PomcCreERT2 mice (Berglund et al J Clin. Invest. 123, 5061–5070 , 2013) were generated on a C57BL/6J background and were kindly provided by Joel K Elmquist. The line was crossed with either ROSA-tdTomato (#:007908, Jackson Laboratory, B6;129S6 mixed genetic background) or ROSA-ZsGreen (#:007906, Jackson Laboratory, C57BL/6J background) lineage-tracking mice. PomcCreERT2 mice were also crossed with Pomc-eGFP mice (Jackson Laboratory, #009593, C57BL/6J background) to generate double reporter PomcCreERT2;tdTomato;Pomc-eGFP animals. The generation of PomcDre mice is described in Biglari et al Nat Neurosci. 2021 Jul;24(7):913-929. This model was backcrossed to a C57BL/6N background and subsequently crossed with ROSA-ZsGreen animals. The age of the mice is indicated in figure legends.<br>Tamoxifen (TAM) was administered at 12 weeks of age in all experiments except Figure S1G, where animals were treated with TAM at 5 weeks of age.<br>Mice were individually housed in standard plastic rodent cages, maintained at 22 ± 2°C on a 12-hour light-dark cycle (lights off at 13:00h) at 45-65% humidity.
Wild animals	No wild animals were assessed in this study
Reporting on sex	The effects of sex on Ghost cell recruitment are shown in Figure S1A. All other studies were performed in male mice
Field-collected samples	No field collected samples were assessed in this study
Ethics oversight	All experiments were conducted in strict compliance with the European Union recommendations (2013/63/EU) and were approved by the French Ministry of Higher Education, Research and Innovation and the local ethical committee of the University of Bordeaux

Note that full information on the approval of the study protocol must also be provided in the manuscript.

Plants

Seed stocks	*Report on the source of all seed stocks or other plant material used. If applicable, state the seed stock centre and catalogue number. If plant specimens were collected from the field, describe the collection location, date and sampling procedures.*
Novel plant genotypes	*Describe the methods by which all novel plant genotypes were produced. This includes those generated by transgenic approaches, gene editing, chemical/radiation-based mutagenesis and hybridization. For transgenic lines, describe the transformation method, the number of independent lines analyzed and the generation upon which experiments were performed. For gene-edited lines, describe the editor used, the endogenous sequence targeted for editing, the targeting guide RNA sequence (if applicable) and how the editor was applied.*
Authentication	*Describe any authentication procedures for each seed stock used or novel genotype generated. Describe any experiments used to assess the effect of a mutation and, where applicable, how potential secondary effects (e.g. second site T-DNA insertions, mosiacism, off-target gene editing) were examined.*
